# Biocatalytic Synthesis of D-Allulose Using Novel D-Tagatose 3-Epimerase From *Christensenella minuta*

**DOI:** 10.3389/fchem.2020.622325

**Published:** 2020-12-10

**Authors:** Yang Wang, Yuvaraj Ravikumar, Guoyan Zhang, Junhua Yun, Yufei Zhang, Amreesh Parvez, Xianghui Qi, Wenjing Sun

**Affiliations:** ^1^School of Life Science, Jiangsu University, Zhenjiang, China; ^2^School of Food and Biological Engineering, Jiangsu University, Zhenjiang, China

**Keywords:** D-tagatose 3-epimerase, D-allulose, *Christensenella minuta*, biochemical characterization, biocatalysis

## Abstract

D-allulose, which is one of the important rare sugars, has gained significant attention in the food and pharmaceutical industries as a potential alternative to sucrose and fructose. Enzymes belonging to the D-tagatose 3-epimerase (DTEase) family can reversibly catalyze the epimerization of D-fructose at the C3 position and convert it into D-allulose by a good number of naturally occurring microorganisms. However, microbial synthesis of D-allulose is still at its immature stage in the industrial arena, mostly due to the preference of slightly acidic conditions for Izumoring reactions. Discovery of novel DTEase that works at acidic conditions is highly preferred for industrial applications. In this study, a novel DTEase, DTE-CM, capable of catalyzing D-fructose into D-allulose was applications. In this study, a novel DTEase, DTE-CM, capable of catalyzing D-fructose into D-allulose was DTE-CM on D-fructose was found to be remarkably influenced and modulated by the type of metal ions (co-factors). The DTE-CM on D-fructose was found to be remarkably influenced and modulated by the type of metal ions (co-factors). The 50°C from 0.5 to 3.5 h at a concentration of 0.1 mM. The enzyme exhibited its maximum catalytic activity on D-fructose at pH 6.0 and 50°C from 0.5 to 3.5 h at a concentration of 0.1 mM. The enzyme exhibited its maximum catalytic activity on -fructose at pH 6.0 and 50°C with a *K*_*cat*_/*K*_*m*_ value of 45 mM^−1^min^−1^. The 500 g/L D-fructose, which corresponded to 30% conversion rate. With these interesting catalytic properties, this enzyme could be a promising candidate for industrial biocatalytic applications.

## Introduction

Rare sugars, ubiquitously found in nature as part of carbohydrate metabolism, are renowned for having a wide range of physiological functions. Many rare sugars possess 70–90% sweetness of sucrose and their low-calorie value allows these biomolecules to be valued by food industries (Park et al., 2016). As each rare sugar exerts its unique health benefits, the quest for many novel ways to produce rare sugars has become a key research area in the current decade. This has become particularly significant for the pharmaceutical sectors, where rare sugars like D-allulose (D-ribo-2-hexulose or D-psicose) has high market price and therapeutic value. D-allulose is a C-3 epimer of D-fructose having 70% sweetness and 0.3% energy relative to sucrose (Matsuo et al., 2001). Some of the notable physiological properties of D-allulose are: (1) anti-hyperglycemic effects (Hossain et al., 2011, 2015), (2) anti-hyperlipidemic effects (Matsuo et al., 2001; Afach et al., 2008), (3) anti-inflammatory effects (Oh, 2007), (4) neuroprotective effects (Takata et al., 2005), (5) reactive oxygen species (ROS) scavenging activity scavenging activity (Suna et al., 2007). Besides its therapeutic potential, D-allulose is also used as food flavor and processing since it reduces the oxidation process greatly (Sun et al., 2004). Inspired by these beneficial properties, the properties, the United States of Food and Drug Administration (USFDA) have included D-allulose in the list of Generally Regarded as Safe Substances (GRAS) which opens a new horizon for many health care industries to supply -allulose as a therapeutic agent (Zhang et al., 2017).

**Figure 1:**
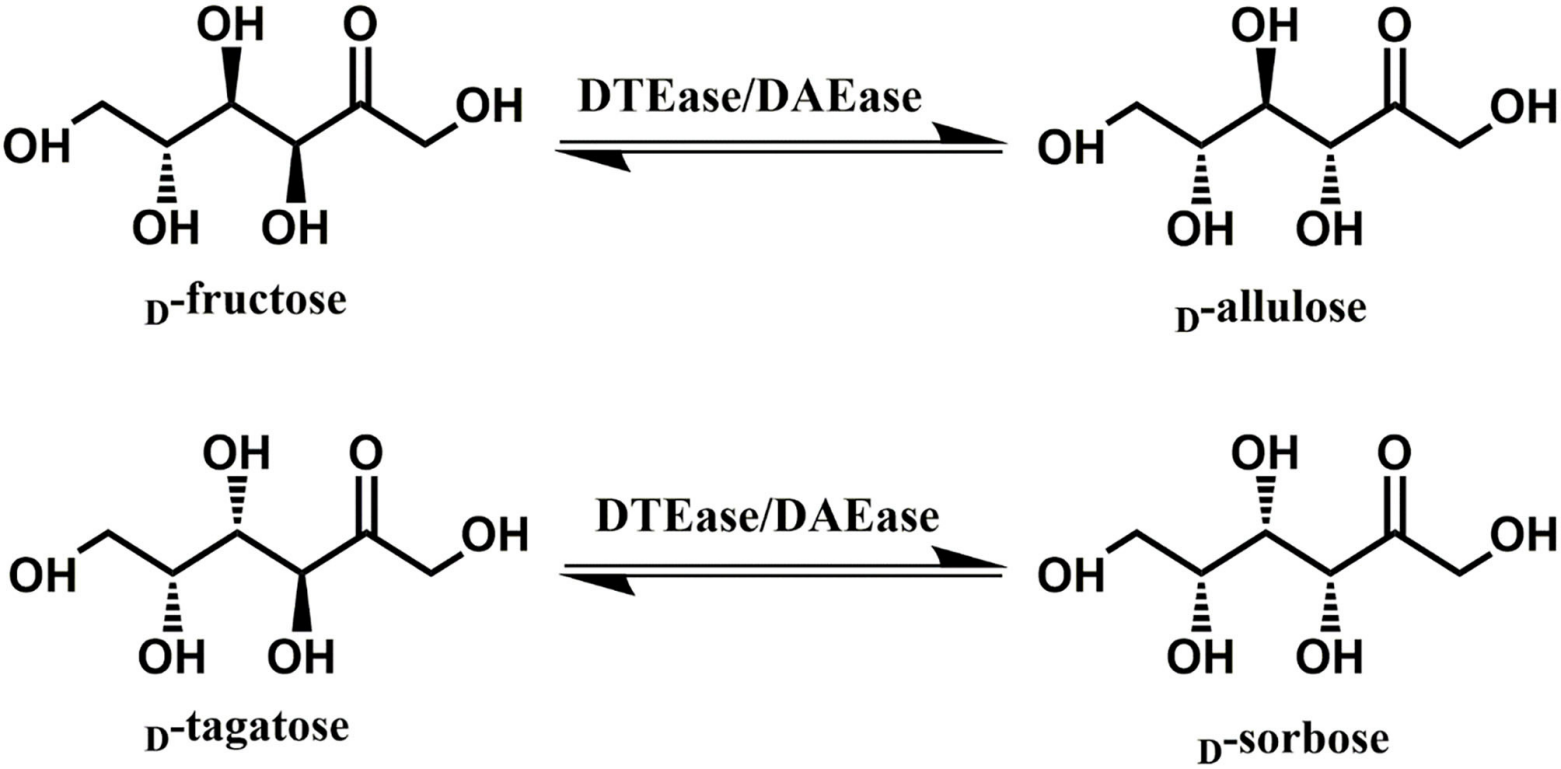
The epimerization reactions of D-fructose and D-tagatose catalyzed by DTEase family.

Since D-allulose is scarcely present in nature, various methods for its large-scale production have been established over the past few decades. Chemical synthesis of D-allulose being laborious and expensive (Emmadi and Kulkarni, 2014), utilizes molybdate ion catalyst (Bilik and Tihlarik, 1973) or 1,2:4,5-di-*O*-isopropylidene-β-D-fructopyranose (McDonald, 1967) for D-allulose synthesis from D-fructose. Some major disadvantages of the chemical routes are the need for complex processing conditions, high catalyst concentration, hazardous side reactions, and non-ecofriendly (Zhang et al., 2020b). Due to these reasons, the use of enzymes as biocatalysts has received impeccable attention in industries. industries. Among such enzymes, D-tagatose 3-epimerase (DTEase) family including D-Tagatose-3-epimerases (DTEases, EC 5.1.3.31) having substrate specificity to D-tagatose and D-allulose 3-epimerases (DAEase, EC 5.1.3.30) with substrate specificity for D-allulose have turned up as the most efficient biocatalysts for D-allulose (Kim et al., 2006a; Zhang et al., 2016). The DTEase family enzymes, being metal-dependent, are capable of catalyzing reversible epimerization of epimerization of ketohexoses at C-3 position to convert D-fructose to D-allulose and D-tagatose to D-sorbose (Figure 1), respectively. Although the genes of DTEase family enzymes are widely predicted in various microorganisms, to date only 19 enzymes of this family are identified and characterized (Patel et al., 2020). DTEases *Pseudomonas cichorii* (Itoh et al., 1994), *Rhodobacter sphaeroides* (Zhang et al., 2009), *Caballeronia fortuita* (Li et al., 2019), and *Sinorhizobium* sp. (Zhu et al., 2019b) and DAEase from *Agrobacterium tumefaciens* (Kim et al., 2006a), *Clostridium cellulolyticum* H10 (Mu et al., 2011) are some of the notable examples. However, there remains a pertinent demand for the robust DTEases/DAEase that can efficiently convert D-fructose to -allulose under acidic pH and at higher temperatures (Bhosale et al., 1996; Zhang et al., 2015). To circumvent non-enzymatic reactions and by-product formation, DTEase/DAEase that work under slightly acidic conditions are highly preferred (Zhang et al., 2015). Further, poor thermal activity of enzymes at higher temperatures (>50°C) necessitates the identification of more novel DTEase that are suited for industrial settings. More importantly, DTEase thermal stability has become a hot issue in the last 10 years after Kim and co-workers demonstrated an improved thermostability in *A. tumefaciens* DAEase attained by protein engineering methods (Kim et al., 2010). Although methods-like-directed evolution and site-directed mutagenesis can generate thermostable enzymes, at times the mutations can lead to poor catalytic activity. Hence, the discovery of new enzymes that has intrinsic desired properties has always been quite useful and identification of such enzymes can be achieved via sequence-based screening of metagenomic libraries or database mining.

Currently, with the abundance of sequence data available, it is quite achievable to mine for DTEase from less investigated sources and environments by bioinformatic analysis. In this study, we identified a potential putative DTEase in the genome of *Christensenella minuta* DSM 22607 (DTE-CM). The gene encoding for DTE-CM was cloned and overexpressed in *E. coli* Rosetta (DE3). The newly identified DTE-CM was successfully used in reaction for the biosynthesis of D-allulose. D-allulose. Remarkably, the obstacle of producing D-allulose under mild acidic conditions could be novel DTE-CM at laboratory conditions.

## Materials and Methods

### Bacterial Strains and Materials

*Escherichia coli* DH5α and Rosetta (DE3) were obtained from the Stratagene, USA and used as the host strains for DNA cloning and expression, respectively. They were routinely cultured in Luria-Bertani (LB) medium containing Kanamycin (50 μg/mL) in a rotary shaker at 37°C and 200 rpm. The plasmid pANY1 was obtained from the Shenyang Agricultural University, China (Gao et al., 2018; Liu et al., 2018), and was used as the expression vector.

The PCR master mix, restriction endonuclease One-Step Cloning Kit, Fast Pure Plasmid Mini Kit and Fast Pure Gel DNA Extraction Mini Kit were purchased from the Vazyme (Nanjing, China). The Ni^2+^-NTA resin for protein purification, were purchased from Sangon Biotech Co., Ltd (Shanghai, China). Electrophoresis reagents were purchased from Bio-Rad (Hercules, CA, USA). Isopropyl β-D-1-thiogalactopyranoside (IPTG), analytical and HPLC grade chemicals used for enzyme assays and characterization were obtained from the Aladdin (Shanghai, China) and the Sinopharm Chemical Reagent (Beijing, China).

### Gene Mining, Cloning, and Expression

Based on the reported DTEase, sequences of DTEase from *R. sphaeroides* were chosen and used as query for mining new DTEase sequences. Sequences having identity between 30 and 70% to query were retrieved using the Blastp search tool in the NCBI database. The retrieved sequences were further processed to determine redundancy and were removed based on the similarity to earlier reported DTEases family enzymes. Putative DTEase gene of *C. minuta* strain showing maximum identity (42.9%) with *R. cellulolyticum* and *C. bolteae* was selected for further cloning process. The gene encoding for DTE-CM was obtained from the complete genome found in GenBank (NCBI accession number: NZ_CP029256.1). The full-length nucleotide sequence of the putative DTEase coding gene was synthesized by Genewiz (Suzhou, China) and then the forward (5′-ACATCAGCCGTAGGATCCATGAAATACGGTTTATTGTACCATTATT-3′) and the reverse primer (5′-GGGGACGTCGACTCTAGACTAGTGATGATGATGGTGATGCG-3′) were designed to introduce homologous arm sequence (underlined) to clone the gene with a sequence for a C-terminal 6 × His tag into pANY1 vector by the Seamless Cloning technology and named as pANY-*Cm-dte*. The plasmid was then transformed into *E. coli* Rosetta (DE3) cells by heat shock method and the transformants were cultured in 0.5 L LB-containing kanamycin (50 μg/mL) at 37°C, 200 rpm. After cells reaching OD_600_ at 0.6–0.8, enzyme expression was induced by adding 1 mM of IPTG (Yun et al., 2018). The culture was then incubated overnight at 25°C and 150 rpm. After the induction period, cells were harvested and washed by centrifuging at 8,000 rpm for 8 min at 4°C.

### Purification of the Recombinant DTE-CM

To purify the recombinant DTE-CM, the centrifuged cell pellets were washed twice with 0.85% NaCl solution and resuspended in lysis buffer (50 mM sodium phosphate buffer, 150 mM NaCl, 1 mM DTT, pH 7.0), and disrupted by sonication at 4°C for 15 min (400 W; 1.5 s working time, 2 s interval) using a sonicator (Sonics & Materials, Newtown, CT, USA). After sonication, the cell lysates were centrifuged at 4°C at 8,000 rpm for 30 min to remove the cell debris, and the supernatant was filtered through a 0.22 μm filter. The filtrate was then loaded onto a Chelating Sepharose Fast Flow resin column (1.0 cm × 10.0 cm), containing Ni^2+^, and allowed to equilibrate with a binding buffer (50 mM sodium phosphate buffer, 500 mM NaCl, 1 mM DTT, pH 7.0). The unbound proteins were eluted from the column with a washing buffer (50 mM sodium phosphate buffer, 500 mM NaCl, 50 mM imidazole, 1 mM DTT, pH 7.0). Then, the recombinant DTE-CM was eluted from the column using an elution buffer (50 mM sodium phosphate buffer, 150 mM NaCl, 300 mM imidazole, 1 mM DTT, pH 7.0). The active fractions were pooled, desalted, and concentrated using an Amicon Ultra 15 system (Millipore) against 50 mM sodium phosphate buffer (pH 7.0). The protein purity was determined by sodium dodecyl sulfate polyacrylamide gel electrophoresis (SDS-PAGE) analysis. The protein concentration was determined by the Bradford assay by using bovine serum albumin as the standard (Bradford, 1976). Thereafter, the purified enzyme was used for the characterization experiments and to determine its kinetic and catalytic properties.

### Enzyme Assay

The enzyme activity of DTE-CM was determined as reported previously (Mu et al., 2011). Briefly, the enzyme reaction was carried out in sodium phosphate buffer (50 mM, pH 6.0) containing Ni^2+^ (1 mM), D-fructose (50 g/L), and enzyme (0.5 μM) in a final volume of 1 mL at 50°C for 10 min. The reaction was stopped by incubating the sample in boiling water (100°C). The reaction samples were centrifuged at 13,000 rpm for 4 min and the clear supernatant was collected and analyzed in HPLC. One unit of enzyme activity was defined as the amount of enzyme catalyzing the formation of 1 μmol D-allulose per minute at pH 6.0 and 50°C.

### Effects of Various Metallic Ions on the Activity of DTE-CM

Effects of metal ions on DTE-CM activity were determined by doing experiments in the presence of various metal ions (Ca^2+^ Co^2+^, Mn^2+^, Mg^2+^, Fe^2+^, Ni^2+^, Zn^2+^, and Cu^2+^) with 1 mM concentration for each. After every mixture was incubated at 4°C for 30 min, the relative enzyme activity was assessed based on the production of D-alluose under standard conditions. The activity obtained with Ni^2+^ was expressed as 100%.

### Effects of Temperature and pH on the Activity of DTE-CM

The influences of temperature on the enzyme activity were analyzed by doing the experiments in phosphate buffer (50 mM, pH 6.0) under various temperatures, ranging from 30 to 80°C. Thermal stability of the enzyme was evaluated by measuring the residual activities of DTE-CM in phosphate buffer (50 mM, pH 6.0) incubated at 40 to 60°C for different time intervals. Similarly, to study the effects of pH on the enzyme activity, enzyme assay was performed at different pHs (5.0–9.0). Different buffers used were acetate buffer (50 mM pH 5.0–5.5), phosphate buffer (50 mM pH 6.0–7.5), and Tris–HCl buffer (50 mM, pH 8.0–9.0). For the pH stability analysis, the purified enzyme was incubated at pH 5.0–8.0 at 4°C for up to 2 h, and its activity was measured under the standard assay conditions. Sample without incubation was used as the control for temperature assays and the original activity at individual pH was taken as 100%.

### Substrate Specificity and Kinetic Properties of DTE-CM

Kinetic parameters for DTE-CM were studied at pH 6.0 and 50°C, using a pseudo-one substrate kinetic model as described elsewhere (Li et al., 2019). Initial rate measurements were done by using enzyme assay in the reaction mixture with varying concentrations of D-fructose, D-allulose, D-tagatose and D-sorbose substrates and Ni^2+^ (1 mM) in phosphate buffer in a total reaction volume of 1 mL. The kinetic parameters for different substrates, such as Michaelis-Menten constant (*K*_*m*_), turnover number (*K*_*cat*_), and catalytic efficiency (*K*_*cat*_*/K*_*m*_) values were obtained using the Lineweaver–Burk plot equation. Kinetic values reported here were attained using three independent experiments.

### Biocatalytic Conversion of _D_-fructose Using DTE-CM

Enzymatic synthesis of D-allulose was carried out in 50 mL of 0.5 L shake flask contained 100, 300, 500 g/L of D-fructose, and 5 mM DTE-CM under the optimum conditions. The progress profile of the enzymatic conversion was every 20 min until the reaction reached equilibrium. The generated products were further subjected to HPLC analysis.

### Analytical Methods

The concentrations of D-fructose, D-allulose, D-tagatose, and D-sorbose were analyzed in HPLC equipped with a refractive index detector (RID-20A, Shimadzu, Japan). Standard HPLC analysis was carried out by using the Ca^2+^-carbohydrate column (300 × 7.7 mm) (HPLX-Ca, Agilent USA) with 20 μL sample injections (Zhang et al., 2020a). The samples were eluted by using milli pore pure water at a flow rate of 0.6 mL/min and 84°C.

## Results and Discussion

### Gene Selection and Sequence Analysis of DTE-CM

**Figure 2:**
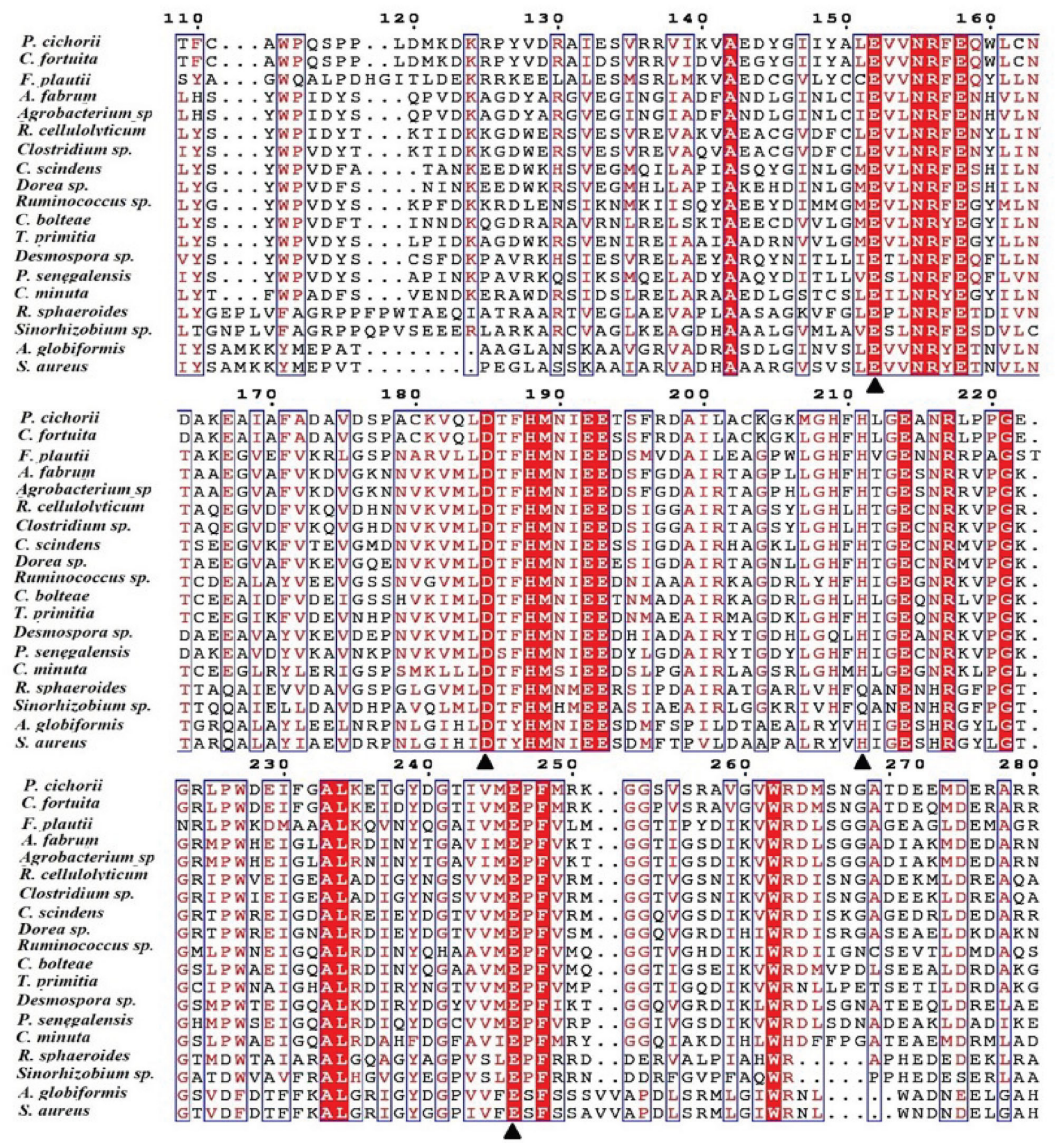
Alignment of the amino acid sequences of DTEase family. The origins of DTEase family and their GenBank Nos are as follows: *P. cichorii* (BAA24429.1); *R. sphaeroides* (ACO59490.1); *C. fortuita* (WP_061137998.1); *Sinorhizobium* sp. (WP_069063284.1); *A. tumefaciens* (AAK88700.1); *C. cellulolyticum* H10 (ACL75304); *Ruminococcus* sp. (ZP_04858451); *Clostridium sp*. (WP_014314767.1);*C. scindens* (B0NGC5); *Desmospora* sp. (F5SL39); *C. bolteae* (A8RG82); *Dorea* sp. (CDD07088.1); *T. primitia* (WP_010256447.1); *F. plautii* (EHM40452.1); *A. globiformis* (BAW27657.1); *Agrobacterium* sp. (EGL65884.1); *P. senegalensis* (WP_010270828.1); *S. aureus* (SQA09501.1); *DaeM* (MN337631); *C. minuta* WP_066519968.1. The residues involved in the metal coordinating site (▴) are symbolized according to the crystal structures of *A. tumefaciens* DAEase (Kim et al., 2006b), *C. cellulolyticum* DAEase (Chan et al., 2012) and *P. cichorii* DTEase (Yoshida et al., 2007), The alignment was prepared using the program ESPript.

**Figure 3:**
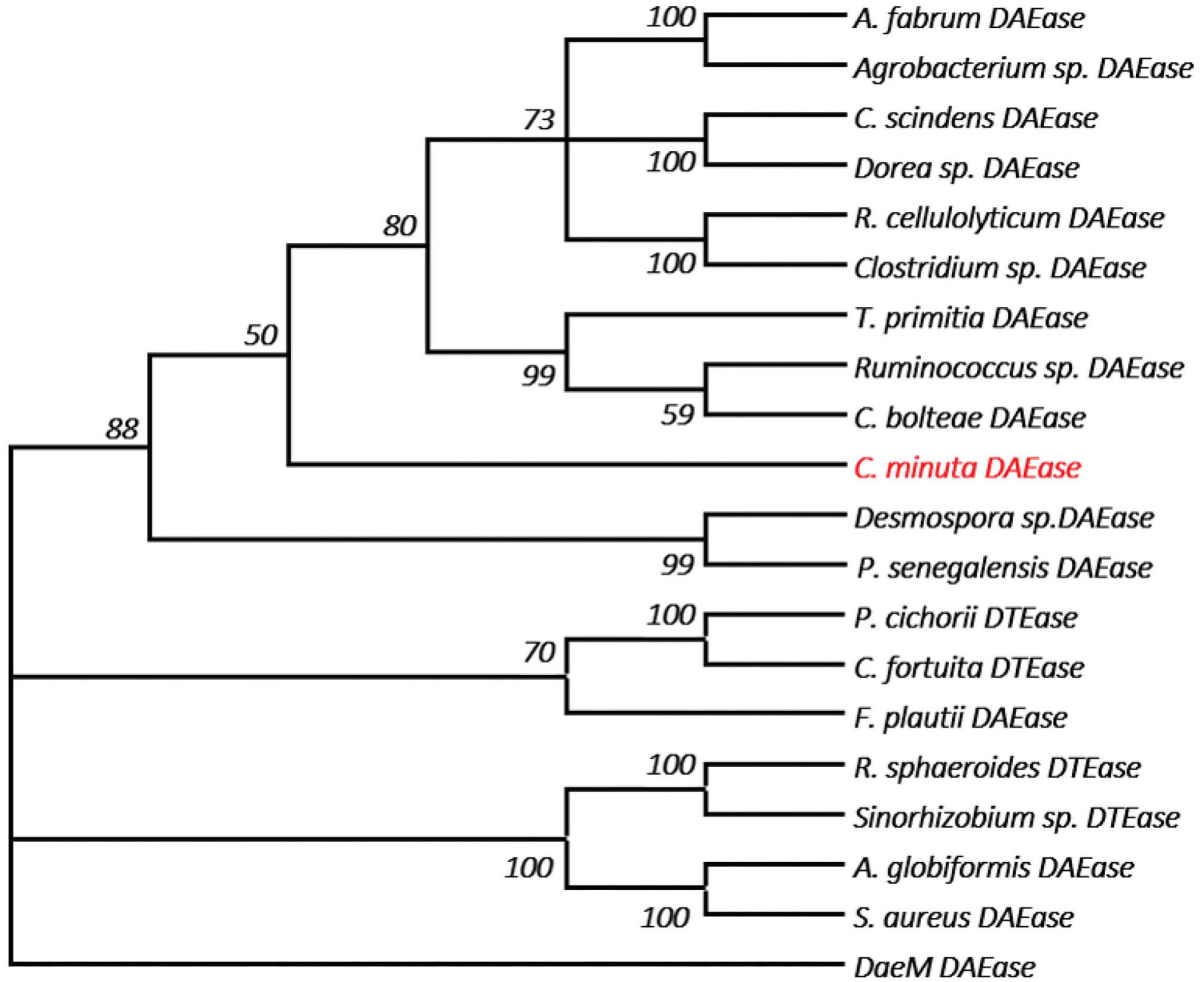
The phylogenetic tree of DTEase family, the microorganism origins of DTEase family with GenBank No.s as follows: *P. cichorii* (BAA24429.1); *R. sphaeroides* (ACO59490.1); *C. fortuita* (WP_061137998.1); *Sinorhizobium* sp. (WP_069063284.1); *A. tumefaciens* (AAK88700.1); *C. cellulolyticum* H10 (ACL75304); *Ruminococcus* sp. (ZP_04858451); *Clostridium sp*. (WP_014314767.1); *C. scindens* (B0NGC5); *Desmospora* sp. (F5SL39); *C. bolteae* (A8RG82); *Dorea* sp. (CDD07088.1); *T. primitia* (WP_010256447.1); *F. plautii* (EHM40452.1); *A. globiformis* (BAW27657.1); *Agrobacterium* sp. (EGL65884.1); *P. senegalensis* (WP_010270828.1); *S. aureus* (SQA09501.1); *DaeM* (MN337631); *C. minuta* (This study) AQ.

One of the key factors for progress in the field of D-allulose production through biocatalysts is the discovery of novel DTEases that can convert D-fructose to D-allulose. In general, D-allulose producing enzymes are being generated continually by identification of more novel enzymes via genome mining approach. Therefore, by using this strategy, it is necessary to find novel DTEases that could produce D-allulose under industrial preferred conditions. In this regard, the reported DTEases having broad substrate specificity and activity toward D-fructose were selected as query sequences. The DTEase from *R. sphaeroides* has been reported for the conversion of D-fructose to D-allulose (Zhang et al., 2009). By using protein sequence of *R. sphaeroides* and KEGG analysis, hypothetical DTEase in *C. minuta*. The human gut bacteria *C. minuta* earned special attention in 2012 when scientists first discovered it as novel genus and species belonging to *Christensenellaceae* family (Morotomi et al., 2012; Goodrich et al., 2014). Being an anaerobic intestinal microbiota with major function in carbohydrate metabolism, it can be expected that *C. minuta* has inherent DTEase family enzymes related to Izumoring strategy (Granström et al., 2004). To identify the DTEase genes in *C. minuta*, phylogenetic analysis was performed with reported DTEase gene family, which exhibited the presence of hypothetical DTEase protein (WP_066519968.1). Multiple sequence alignment (MSA) of DTE-CM with the reported DTEase family candidates revealed that motifs such as metal coordinating sites Glu150, Asp183, His209, and Glu244 were well-conserved (Figure 2). DTE-CM is homologous with DTEase family having 42.9% sequence identity with *C. cellulolyticum* DAEase (Mu et al., 2011), *C. bolteae* DAEase (Jia et al., 2014), and 20% identity to *S. aureus* (Zhu et al., 2019a), respectively. In addition, the phylogenetic tree obtained by neighbor-joining method reveals the presence of putative DTEase within single gene cluster of reported DTEase family, implying the likelihood of having conserved functional motifs responsible for D-fructose epimerization (Figure 3). Overall, the conserved aspect of DTEase motifs responsible for D-fructose epimerization (Figure 3). Overall, the conserved aspect of DTEase

### Heterologous Expression of DTE-CM Gene and Purification of Recombinant DTE-CM

**Figure 4:**
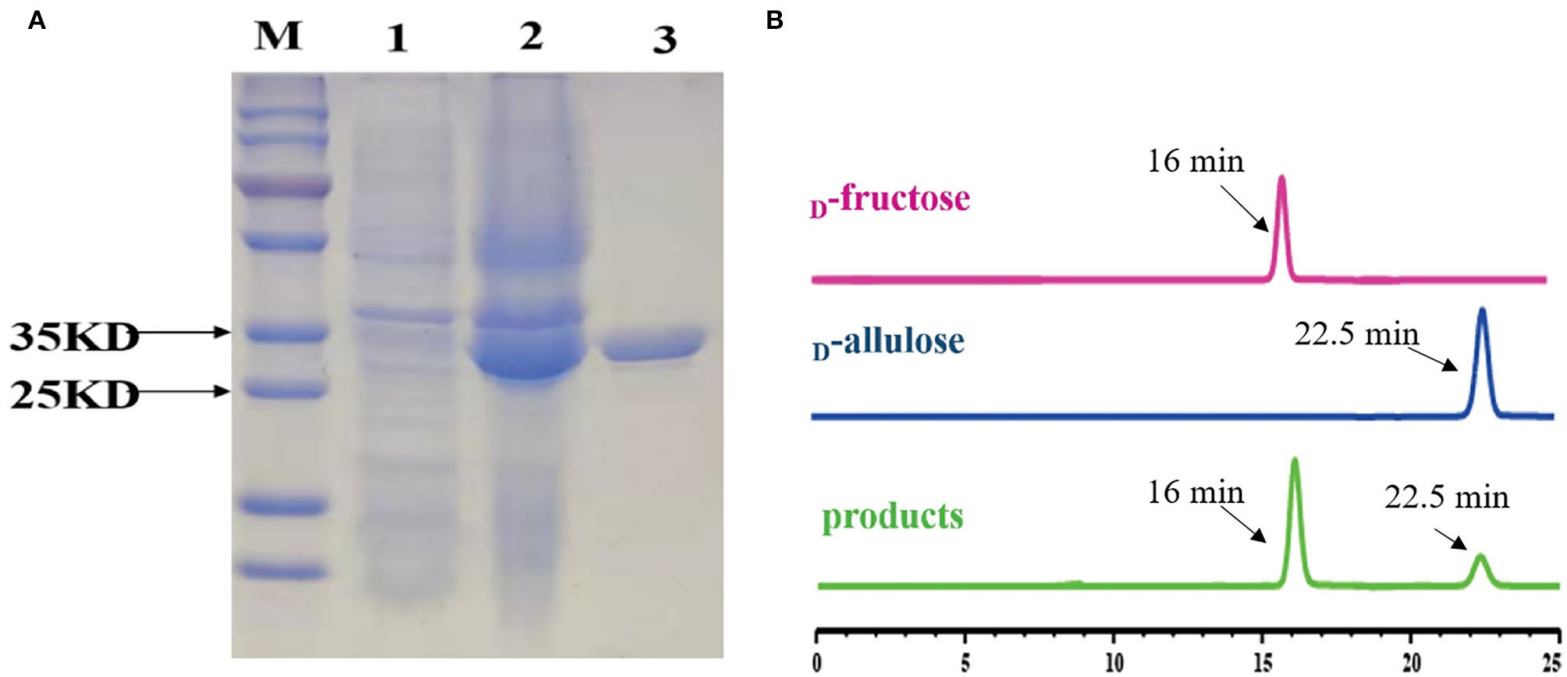
SDS-PAGE analysis of the DTE-CM and HPLC analysis of the isomerization products by the purified DTE-CM. **(A)** SDS-PAGE analysis. Lane M, protein marker; Lane 1, the control *E. coli* Rosetta (DE3) cells harboring plasmid pANY1; Lane 2, *E. coli* Rosetta (DE3) harboring pANY-*Cm*-*dte*; Lane 3, purified recombinant DTEase. **(B)** The HPLC map of DTE biotransformation sample compared with standard D-allulose. Peak 1 is D-fructose; peak 2 is D-allulose; peak 3 is the isomerization products.

To functionally characterize the target enzyme, the gene *dte* of *C. minuta* (GenBank accession No: WP_066519968.1) was synthesized and cloned into the pANY1 expression vector to set up IPTG-inducible gene expression in *E. coli* Rosetta (DE3) cells. The 6×His tag was added at the carboxy-termini to protein for the affinity-based enzyme purification. Cultures that reached log-phase (OD_600_ 0.6–0.8) were treated with 1 mM IPTG to induce DTEase expression at 25°C and 150 rpm. Then, expression was carried out overnight. The cells were then harvested and subjected to the purification process. However, the enzyme becomes cloudy and precipitated in large quantities after passing through the Ni^2+^-NTA affinity column. This might be due to the protein denaturation. Hence, 1 mM DTT was added to the lysis buffer and further purification provided the enzyme in an active form. After the purification process, expression levels and purity were assessed by SDS-PAGE analysis. As shown in Figure 4A, the recombinant protein was well-expressed and appeared in a single band at ~33 kDa. The purified DTE-CM was subsequently used for the enzymatic characterization. Further, ability of the purified enzyme to isomerize D-fructose for D-allulose was confirmed by the HPLC analysis. The appearance of peak at 16 min corresponded to D-fructose and 22.5 min to D-allulose (Figure 4B). This result proved that DTE-CM can successfully convert D-fructose to D-allulose, which encourage us characterize its biochemical parameters.

### Effects of Metal Ions on DTE-CM Activity

**Figure 5:**
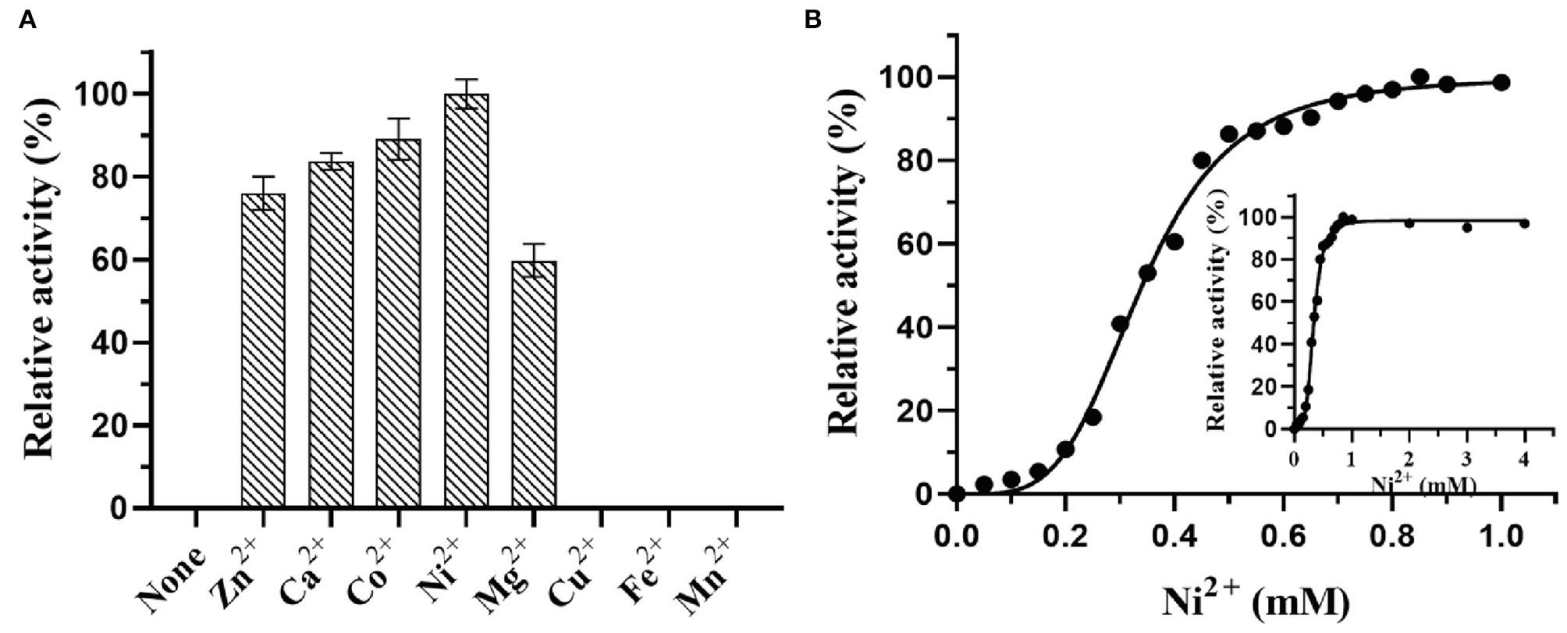
Effect of metal ions **(A)** and metal ion concentration **(B)** on DTE-CM activity.

In most of the DTEases reported to date, metal ions play a vital role in isomerizing monosaccharides. Besides the catalytic activity, metal ions also influence the stability of the enzymes. Thus, to interrogate the effect of a wide range of physiologically active metal ions, the purified enzyme was tested for the activity in the presence and absence of different divalent metal ions. The final concentration of 1 mM metal ions was used for the analysis. The relative catalytic activity with and without metal ions were determined by measuring the maximum epimerization of D-fructose. The results reveal that presence of Cu^2+^, Fe^2+^, and Mn^2+^ led the enzyme to become inactive, whereas the Ni^2+^ (100%) and Co^2+^ (83%) showed significant epimerization activity toward D-fructose. Moreover, Mg^2+^, Zn^2+^, and Ca^2+^ ions also showed moderate catalytic activity as represented in Figure 5A. Surprisingly, DTE-CM did not exert any activity in the absence of metal cofactor. Furthermore, addition of metal ion chelator, EDTA (10 mM) into the reaction mixture caused a loss in the enzyme activity exceptionally. These results showed that, like other reported DTEases, DTE-CM was a metal-dependent enzyme and required Ni^2+^ for improving its catalytic efficiency. Also, it is noteworthy that, DTE-CM was the first DTEase with Ni^+2^ as the highly influencing metal cofactor, which was different from other DTEases that required Co^2+^ for *C. cellulolyticum* DAEases (Mu et al., 2011) and *Desmospora* sp. DAEases (Zhang et al., 2013b), Mn^2+^ for *A. tumefaciens* DAEases (Kim et al., 2006a), and *C. scindens* DAEase (Zhang et al., 2013a), Mg^2+^ for *S. aureus* DAEase (Zhu et al., 2019a). With these initial findings, optimum metal ion (cofactor) concentration for DTE-CM was evaluated. Generally, co-factor concentration played an important role in the applications of biocatalyst on large scale. Therefore, to find out the best concentration, a detailed analysis of catalytic activity with respect to Ni^2+^ ion concentration (0–1 mM) was measured. From the results (Figure 5B), it was evident that maximum enzyme activity was found at 1 mM Ni^2+^ and the activity decreased with respect to lower concentration. Furthermore, beyond 1 mM concentration of Ni^2+^, enzyme activity remained stable. However, a slight decrease was noted at much higher concentrations (1−4 mM).

### Effects of Temperature and pH on the Activity and Stability of DTE-CM

**Figure 6:**
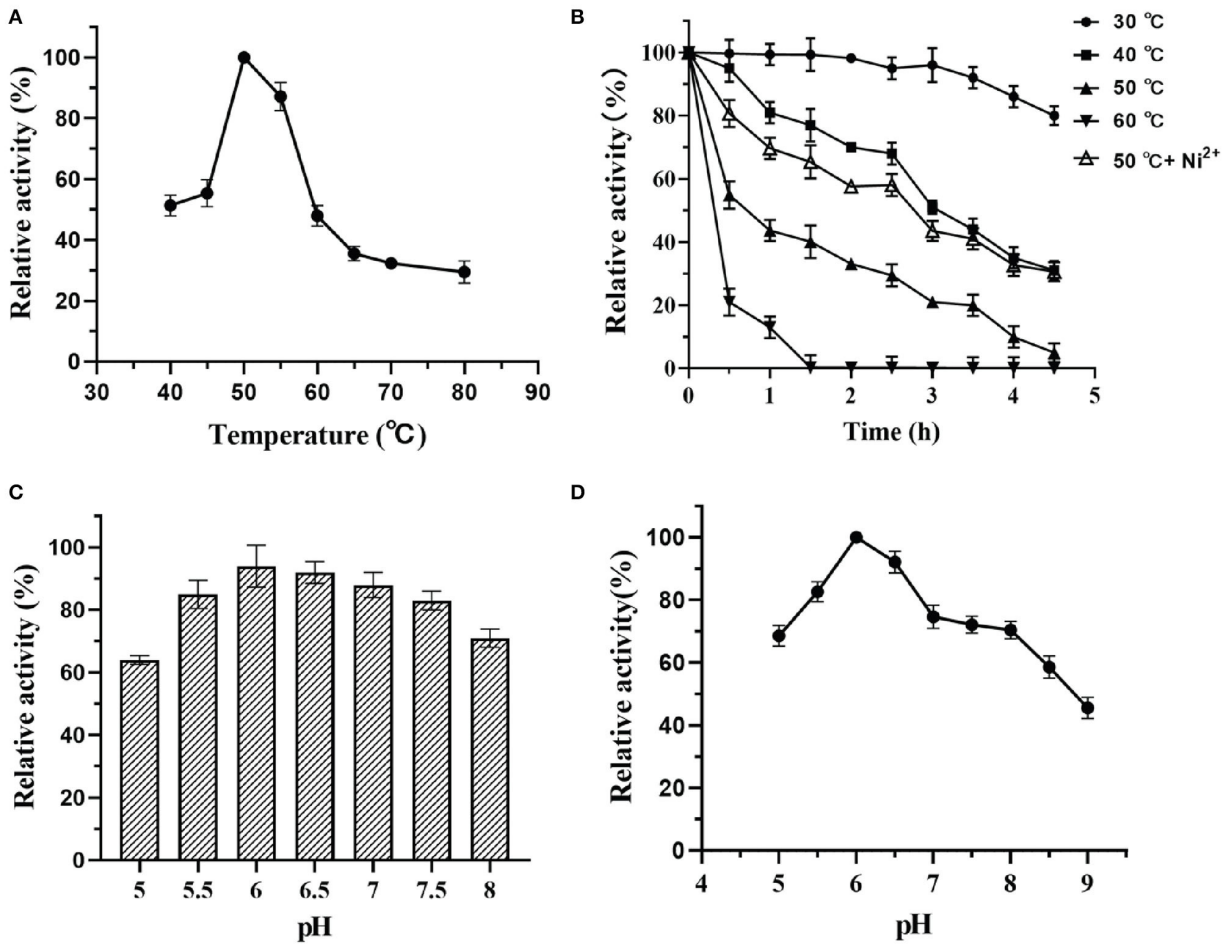
Effect of pH and temperature on activity of DTE-CM: **(A)** temperature dependence; **(B)** pH dependence; **(C)** pH stability; **(D)** thermostability analysis.

The activity and stability of enzymes mainly depend on the factors like pH and temperature that are used in the reaction mixture. Thus, to investigate these parameters, the effects of temperature on the catalytic potential of DTE-CM were examined at temperatures ranging from 40 to 80°C. As shown in Figure 6A, the maximum activity was observed at 50°C, thus indicating the mesophilic nature of the enzyme. At 60°C, the enzyme lost 45% of its activity. The activity further diminishes with respect to an increase in temperature. Less than 30% activity was measured at 70 and 80°C. During thermostability assay, DTE-CM was exposed to a wide range of temperature ranging from 30 to 60°C and its activity were recorded (Figure 6B). After incubation for 4 h, 80% of the residual activity was retained at 30°C, no more than 10% of the residual activity was left at 50°C. Furthermore, complete loss of enzyme activity was observed at 60°C after 1.5 h. Interestingly, addition of 0.1 mM Ni^2+^ exerted 30% higher activity at 50°C. It suggests that metal co-factors play a significant role in attributing the thermal stability to DTE-CM, which were similar to the half-lives of *C. cellulolyticum* DAEase reported at 60°C that increased from 10 to 408 min when incubated with 0.1 mM Co^2+^ (Mu et al., 2011).

Likewise, the effects of pH on the enzyme activity was determined by measuring the epimerization of D-fructose to produce D-allulose. At the optimum temperature, the effect of pH on enzyme was evaluated in a range D-fructose to produce D-allulose. At the optimum temperature, the effect of pH on enzyme was evaluated in a range from 5.0 to 9.0 as depicted in Figure 6C. From the results, it was found that DTE-CM showed a good activity (80% and above) under the acidic conditions (pH 5.5–6.5), where the highest (100%) was recorded at pH 6.0. However, activity started to gradually decline under alkaline conditions. Similar pH optima (pH 6.0) were found in *Dorea* sp. strain CAG317 (Zhang et al., 2015) and the other reported DAEase and DTEase exhibited optimum pH in a range between 7 and 8 Figure 6D, more than 75% activity was retained at pH 5.0–8.0 after incubating the enzyme at 4°C for 2 h, which indicates that the DTE-CM was more stable under slightly acidic and neutral conditions compared to alkaline conditions. It was reported that acidic conditions could greatly reduce the non-enzymatic reactions of carbohydrates and unwanted by-product formation in the biosynthesis of D-allulose (Kim et al., 2006a). This suggests that the DTE-CM of unwanted by-product formation in the biosynthesis of D-allulose (Kim et al., 2006a). This suggests that the DTE-CM of

### Substrate Specificity and Enzyme Kinetics

Enzymes that has strong catalytic property are generally considered as the robust catalysts and such biocatalyst are often preferred in industries. To find the applicability of the DTE-CM in D-allulose production, substrate specificity, and kinetic properties were investigated. Interestingly, DTE-CM showed catalytic activity for all four substrates, and the relative activities on D-tagatose, D-ribulose D-fructose, and D-allulose were found to be 100, 40.2, 68, and 26%, respectively (Table 2). It is noteworthy that the catalytic activity of the reported DTEases on _L_-sorbose have rarely been determined or reported for their undetectable catalytic activity. The best DTE-CM was D-tagatose. Similar substrate preference was also reported for *P. cichorii* DTEase (Itoh et al., 1994), *C. fortuita* DTEase (Li et al., 2019), and *Sinorhizobium* sp. DTEase (Zhu et al., 2019b); While the optimum substrate of *A. tumefaciens* DAEase (Kim et al., 2006a) and *R. sphaeroides* DTEase (Zhang et al., 2009) was D-allulose and D-fructose, respectively (Table 1). The DTE-CM had high relative activity on D-allulose and D-sorbose comparing with the other reported DTEases, which indicate that the broad specificity of this enzyme could be instrumental in the production of rare sugars.

**Table 1:** Comparison of biochemical properties and kinetic parameters of DTEase family enzymes.

**DTEase family**	**Temperature** ** (^**°**^C)**	**pH**	**Metal** ** dependence**	**Metal** ** ions**	**Substrate** ** optimum**	**Half-life** ** (min)**	**Equilibrium**	***K_**cat**_*/*K_**m**_*^**b**^**	**References**
							**Ratio^**a**^**	**(mM^**−1**^min^**−1**^)**	
*R. sphaeroides* DTEase	40	9.0	No	Mn^2+^	D-fructose	NR	23:77 (40°C)	NR	Zhang et al., 2009
*P. cichorii* DTEase	60	7.5	No	Mn^2+^	D-tagatose	60 (50°C)	20:80 (30°C)	NR	Itoh et al., 1994
*C. fortuita* DTEase	65	7.5	No	Co^2+^	D-tagatose	63 (60°C)	29.5:70.5 (65°C)	432.6	Li et al., 2019
*C.minuta* DTEase	50	6.0	Yes	Ni^2+^	D-allulose	40 (50°C)	30:70 (50°C)	124	This study
*Sinorhizobium* sp. DTEase	50	8.0	No	Mn^2+^	D-tagatose	NR	NR	118.2	Zhu et al., 2019b
*A. tumefaciens* DAEase	50	8.0	No	Mn^2+^	D-allulose	64 (50°C)	33:67 (40°C)	205	Kim et al., 2006a
*C. cellulolyticum* DAEase	55	8.0.	Yes	Co^2+^	D-allulose	10 (60°C)	32:68 (40°C)	186.4	Mu et al., 2011
*C.scindens* DAEase	60	7.5	Yes	Mn^2+^	D-allulose	108 (50°C)	28:72 (50°C)	64.5	Zhang et al., 2013a
*Desmospora* sp.DAEase	60	7.5	Yes	Co^2+^	D-allulose	120 (50°C)	30:70 (70°C)	327	Zhang et al., 2013b
*Ruminococcus* sp. DAEase	60	7.5–8.0	No	Mn^2+^	D-allulose	96 (60°C)	28:72 (60°C)	51	Zhu et al., 2012
*C.bolteae DAEase*	55	7.0	Yes	Co^2+^	D-allulose	43 (55°C)	32:68 (60°C)	107	Jia et al., 2014
*Clostridium* sp.DAEase	65	8.0	Yes	Co^2+^	D-allulose	15 (60°C)	28:70 (65°C)	141.4	Mu et al., 2013
*T.primitia* DAEase	70	8.0	Yes	Mn^2+^	D-allulose	30 (50°C)	28:72 (70°C)	144	Zhang et al., 2016
*Dorea* sp. DAEase	70	6.0	Yes	Co^2+^	D-allulose	30 (60°C)	30:70 (70°C)	412	Zhang et al., 2015
*F. plautii* DAEase	65	7.0	Yes	Co^2+^	D-allulose	130 (60°C)	NR	156	Park et al., 2016
*A.globiformisM30* DAEase	70	7.5–8.0	NO	Co^2+^	D-allulose	NR	NR	182.7	Yoshihara et al., 2017
*Agrobacterium* sp. DAEase	55–60	7.5–8.0	Yes	Co^2+^	D-allulose	75 (55°C)	30:70 (55°C)	NR	Tseng et al., 2018
*P.senegalensis* DAEase	55	8.0	No	Mn^2+^	D-allulose	NR	30:70 (55°C)	39	Yang et al., 2019
*S. aureus* DAEase	70	8.0	No	Mg^2+^	D-allulose	NR	NR	NR	Zhu et al., 2019a
*DAEM* DAEase	75–80	70	Yes	Co^2+^	D-allulose	49 (80°C)	31:69 (80°C)	NR	Patel et al., 2020

*NR, not reported*.

a *The Equilibrium ratio was determined between D-fructose and D-allulose under the optimum reaction condition of each enzyme*.

b *The k_cat_/K_m_ determined with D-allulose as substrate*.

The kinetic parameters of DTE-CM with D-fructose, D-allulose, D-tagatose, and D-sorbose were determined (Table 2). The *K*_*m*_ and *K*_*cat*_ values for D-allulose were 53.8 mM and 6,572 min^−1^, respectively. For D-fructose and D-sorbose, the *K*_*m*_ and values were 94.7 mM and 4,230 min^−1^ and 66.8 mM and 5,874 min^−1^, respectively. Additionally, D-tagatose, which showed the highest relative activity also provided the lowest *K*_*m*_ value (34.9 mM) and the highest *K*_*cat*_ value (9,318 min^−1^). The *K*_*cat*_*/K*_*m*_ value of results DTE-CM for D-allulose were compared to a previously reported eosinophilic DAEase from *Dorea* sp. (Zhang et al., 2015), where *K*_*cat*_*/K*_*m*_ was 432.6 mM^−1^min^−1^. Interestingly, our enzyme had a significantly lower *K*_*cat*_*/K*_*m*_ value of 124 mM^−1^min^−1^, showing that DTE-CM was more conducive to the production of D-allulose in the reversible reaction between D-fructose and D-allulose. Furthermore, the *K*_*cat*_*/K*_*m*_ of DTE-CM for D-tagatose (267 mM^−1^min^−1^) was higher than D-allulose (124 mM^−1^min^−1^), which was consistent with other DTEases, except *one* reported from *R. sphaeroides* (Table 1). Taken together, based on the catalytic efficiency and substrate specificity, it suggests that DTE-CM is a novel DTEase.

**Table 2:** Activities and kinetic parameters of DTE-CM on four substrates.

**Substrates**	***K_***m***_*(mM)**	***K_***cat***_*(min^**−1**^)**	***K_***cat***_/K_***m***_*(mM^**−1**^min^**−1**^)**	**Relative activity (%)**
D-fructose	94.7 ± 1.2	4230 ± 34	45 ± 4.5	26 ± 1.8
D-allulose	53.8 ± 3.4	6572 ± 44	124 ± 6.3	68 ± 0.5
D-tagatose	34.9 ± 1.6	9318 ± 36	267 ± 3.1	100 ± 0.4
D-sorbose	66.8 ± 2.6	5874 ± 29	89 ± 2.9	40.2 ± 1.2

*All assays were repeated three times, and the data are shown as mean ± SD*.

### Biological Production of D-Allulose From D-Fructose

**Figure 7:**
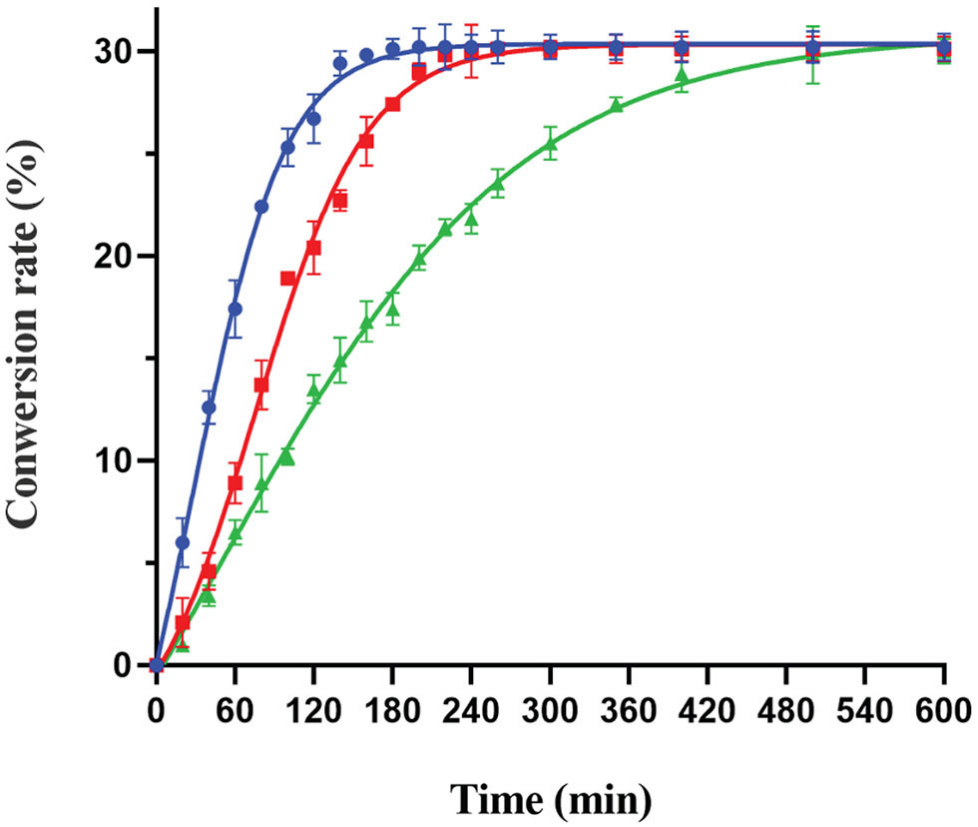
Bioconversion of D-fructose to D-allulose by DTE-CM: 100 (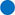); 300 (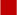); 500 g/L (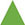).

D-allulose is an important food additive, used as a sugar substitute and flavor enhancer in many food products (Jiang et al., 2020). Recently, the biocatalytic production of D-allulose from D-fructose has gained significant attention as a potential alternative to chemical methods. In order to investigate the capability of DTE-CM producing D-allulose from D-fructose, biotransformation was performed at pH 6.0 and 50°C consisting 10 μM of enzyme and different concentration of D-fructose. When adding 100, 300, and 500 g/L of D-fructose, the enzymatic reaction reached equilibrium at 1.5, 4, and 10 h, respectively, and yielded 30.2, 90.3, and 150 g/L D-allulose, respectively (Figure 7). The equilibrium ratio between D-fructose and D-allulose of the DTE-CM was 30:70 with 30% conversion rate which was the highest under the acidic conditions among DTEases thus far (Table 1). These results indicated that DTE-CM is a potential biocatalyst for producing D-allulose.

## Conclusion

In summary, we have demonstrated application of the intestinal gut bacteria DTE-CM as a novel biocatalyst for D-allulose production. The enzyme evaluated in this work showed broad substrate specificity and optimum activity at acidic conditions (pH 6.0), with good catalytic activity toward D-fructose. The enzyme being metal-dependent showed the acidic conditions (pH 6.0), with good catalytic activity toward D-fructose. The enzyme being metal-dependent showed the highest activity with Ni^2+^ ion, and a lower concentration of Ni^2+^ ions enabled the enzyme to attain improved thermal tolerance. The enzyme exhibited a wide pH and temperature profile with maximum relative activity, showing 30% conversion ratio at pH 6.0 and 50°C. Taken together, this study suggests DTE-CM could be a promising biocatalyst for

## Data Availability Statement

The original contributions presented in the study are included in the article/supplementary materials, further inquiries can be directed to the corresponding author/s.

## Author Contributions

XQ, YW, and WS conceived and designed the experiments. YW carried out the experiments, analyzed the data, and drafted the manuscript. JY, GZ, and YZ assisted in the recombinant strain construction and protein purification. YR, XQ, and AP discussed and revised the manuscript. All authors have read and approved the manuscript.

## Conflict of Interest

The authors declare that the research was conducted in the absence of any commercial or financial relationships that could be construed as a potential conflict of interest.
